# Sensor Location Optimization of Wireless Wearable fNIRS System for Cognitive Workload Monitoring Using a Data-Driven Approach for Improved Wearability

**DOI:** 10.3390/s20185082

**Published:** 2020-09-07

**Authors:** Masudur R. Siddiquee, Roozbeh Atri, J. Sebastian Marquez, S. M. Shafiul Hasan, Rodrigo Ramon, Ou Bai

**Affiliations:** Human Cyber-Physical Systems Laboratory, Florida International University, Miami, FL 33174, USA; ratri001@fiu.edu (R.A.); jmarq056@fiu.edu (J.S.M.); shasa022@fiu.edu (S.M.S.H.); rramo066@fiu.edu (R.R.); obai@fiu.edu (O.B.)

**Keywords:** sensor location optimization, Functional Near-Infrared Spectroscopy (fNIRS), hemodynamics, wireless wearable fNIRS, machine learning, linear SVM, cognitive workload monitoring

## Abstract

Functional Near-Infrared Spectroscopy (fNIRS) is a hemodynamic modality in human cognitive workload assessment receiving popularity due to its easier implementation, non-invasiveness, low cost and other benefits from the signal-processing point of view. Wearable wireless fNIRS systems used in research have promisingly shown that fNIRS could be used in cognitive workload assessment in out-of-the-lab scenarios, such as in operators’ cognitive workload monitoring. In such a scenario, the wearability of the system is a significant factor affecting user comfort. In this respect, the wearability of the system can be improved if it is possible to minimize an fNIRS system without much compromise of the cognitive workload detection accuracy. In this study, cognitive workload-related hemodynamic changes were acquired using an fNIRS system covering the whole forehead, which is the region of interest in most cognitive workload-monitoring studies. A machine learning approach was applied to explore how the mean accuracy of the cognitive workload classification accuracy varied across various sensing locations on the forehead such as the Left, Mid, Right, Left-Mid, Right-Mid and Whole forehead. The statistical significance analysis result showed that the Mid location could result in significant cognitive workload classification accuracy compared to Whole forehead sensing, with a statistically insignificant difference in the mean accuracy. Thus, the wearable fNIRS system can be improved in terms of wearability by optimizing the sensor location, considering the sensing of the Mid location on the forehead for cognitive workload monitoring.

## 1. Introduction

The invention of the wheel introduced a new role for humankind, the operator. This job requires humans to take into account the current state of the system being operated and the current environmental situation the system is in, and to then perform cognitively assessed actuating commands to operate the system effectively and safely. From riding a bicycle to operating an aircraft, these jobs exert various levels of cognitive load on the operator depending on the systems. The safety of the human users accompanying the operator is highly dependent on the continuous cognitive effort of the operator, which is also denoted as the cognitive workload on the operator. For instance, a study on aviation crashes [1] based on 329 major airline crashes claimed that 38% of the crashes were probably caused due to pilot error. Thus, ensuring a balanced and continuous human operator’s cognitive effort via monitoring an operator’s cognitive workload could improve the safety of such mission-critical operations by reducing the probability of human error.

In an effort to improve safety by assessing and balancing operators’ cognitive workload, subjective tests, such as [2] NASA-TLX, have been used widely, which can only be performed after the operator has completed the task and based on his experience during the task. The technological advancement in the modalities named Functional Magnetic Resonance Imaging (fMRI), Electroencephalography (EEG) and Functional Near-Infrared Spectroscopy (fNIRS) in about the last three decades brings a new dimension to cognitive workload monitoring. Now, it can be even monitored while the operator is performing the task. Several studies [3,4,5,6] on cognitive workload assessment have already promisingly showed that physiological signals acquired using fMRI, EEG, and fNIRS or a combination of these modalities [7,8], which are faster than the subjective tests, can be used to monitor cognitive workloads.

The increased neural activity [9] due to the increased cognitive workload increases the oxygen consumption in the cerebral cortex, which is later regulated by the brain’s control mechanism called glial regulation [10]. This blood oxygen-dependent phenomenon can be measured using fMRI [11]. Although fMRI is considered as the de facto standard in detecting cerebral regional blood oxygen concentration change, the technological limitations such as the huge size, high cost, high system complexity, high artefact susceptibility, and low temporal resolution greatly reduce its usability in out-of-the-lab environments. On the other hand, fNIRS is a less expensive technology with higher portability and higher temporal resolution but with a comparable result to fMRI [12,13,14,15,16] in the detection of local cerebral oxygenation changes, although there is some compromise of the signal-to-noise ratio (SNR) and spatial resolution. In the case of EEG application in cognitive workload assessment [5,17,18], though it is capable of a high temporal resolution, it is less portable and less immune to ambient electrical noise. This drawback due to electrical noise is not present in fNIRS due to its optical nature of technological implementation.

**Figure 1:**
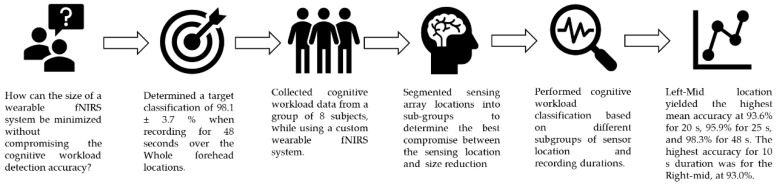
Graphical abstract of the study.

In research into operators’ cognitive workload assessment, fNIRS has successfully been used in various studies in actual or simulated environments, such as car driving [19,20], train driving [21] and flight simulation [22]. The recent studies relating to cognitive tasks [23,24] demonstrated that even wearable fNIRS systems can detect hemodynamic changes associated with the cognitive workload. Moreover, the wearability of the fNIRS system enables such experiments to be conducted in real-life scenarios, such as during walking, driving a vehicle and outdoor navigation [19,23,24,25,26]. These studies are highly influential in the field of engineering applications utilizing the knowledge from cognitive science via fNIRS and also experimentally prove that fNIRS can be implemented in out-of-the-lab situations. All the above experiments were conducted using systems that sensed the whole forehead for cognitive workload detection. In that case, the wearability of the system would significantly degrade the applicability of the modality. In other words, wearing a system on the forehead for several minutes or hours may be acceptable in the case of experiments. However, in the case of the practical application of fNIRS for operators’ cognitive workload detection during their whole-day working period, the wearability of the system with respect to the system dimensions, weight and duration of operation on a single battery recharge are challenges to be taken into account. The research question is how to improve the wearability of fNIRS devices for lengthy cognitive workload monitoring while maintaining the assessment accuracy. In this regard, we hypothesized that the size of a wearable fNIRS system could be minimized while maintaining a high cognitive workload detection accuracy. The graphical abstract of this study is depicted by Figure 1.

During the cognitive load, the portion of the cognitive system that retains the information in the short term, necessary for processing to accomplish the cognitive tasks, is known as the working memory [27], which is widely assumed to be served by the Prefrontal Cortex (PFC) along with central executive function. Several studies related to cognitive function conform with this assumption from the observation of high hemodynamic activities in the PFC during the cognitive workloads [3,9,14,28,29]. This observation of regional cerebral activation during cognitive tasks leads to the implementation of the fNIRS modality in assessing cognitive workload [6,30,31,32] by sensing the PFC optically. These studies focused mainly on anatomically exploring the activation of the brain region by various cognitive processes at deep or shallow depth from the skin. Thus, in such studies, the optimization of sensor locations regarding the wearability of the fNIRS system was not considered. Thereafter, cognitive workload assessment studies focused on fNIRS typically sense the entire front area to identify the workload. To the best of the knowledge of the authors, there is no study addressing this problem of optimization and determining whether it is necessary to sense the whole forehead for the successful detection of cognitive workloads. Furthermore, the compromise of the precision of cognitive workload detection if only a certain smaller portion of the forehead is sensed remains unaddressed.

## 2. Materials and Methods

### 2.1. Participants

Eight healthy volunteers (six males and two females), with no history of neurovascular and cognitive disorders, participated in this study. The study was approved by the Institutional Review Board (IRB-19-0091) of Florida International University, and signed informed consent was obtained from all the subjects prior to the study. 

### 2.2. Experimental Design

**Figure 2:**
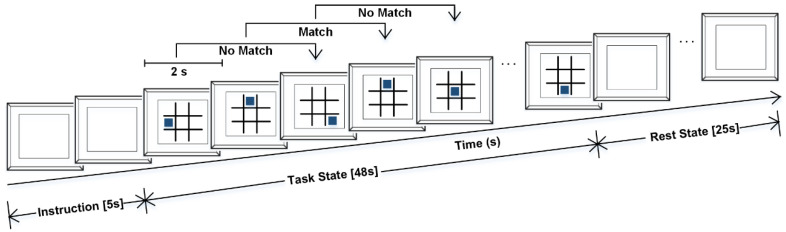
Positional 2-back test. Each event is 2 s long, and the task state lasts for 48 s. Afterward, a 25 s Rest state followed, when the subjects did not move and remained visually affixed to the blank computer screen.

To induce a cognitive workload in human subjects, the n-back task is a widely used [6,7,28] paradigm related to cognitive study in research, which was first demonstrated by Kirchner, W.K. [33]. In this paradigm, the subject observes a series of events during the testing period. If any of the events match with n-events before then, the subject provides feedback, where n could be 1, 2, 3 and so forth, depending on the requirement. As the target of this study is to explore how the detection accuracy for cognitive workload varies due to the sensing location on the human forehead, a moderate level of cognitive workload induction was applied, which is assumed to be represented by the 2-back test [34]. A free, open-source piece of software [35] named “Brain Workshop” was used to simulate the positional 2-back task, where a solid colored square changed its position randomly within a 3-by-3 grid every two seconds (Figure 2 depicts this task paradigm). If the current position of the square matched its position two events before, the subject pressed a button on the keyboard, and they did nothing if the position did not match. In each session of recording, there were 24 events of the positional 2-back task, which spanned 48 s, and afterward, there was 25 s of relaxing, when the subjects did nothing. This relaxing period was considered as the Rest state [7]. The recording of each session started 10 s prior to the start of the 2-back task, and this 10 s was used as the baseline of the diffused optical signal in the conversion of the absorption of optical signals to hemodynamic change. Each subject did 10 such sessions, where the subjects relaxed for about 30 s between the sessions, and during these periods, data were not recorded and the subjects were free to move. The sessions with less than 90% accuracy for 2-back task performance were rejected during the recording [7], assuming less cognitive involvement of the subject in the task.

### 2.3. Data Acquisition and Signal Processing

**Figure 3:**
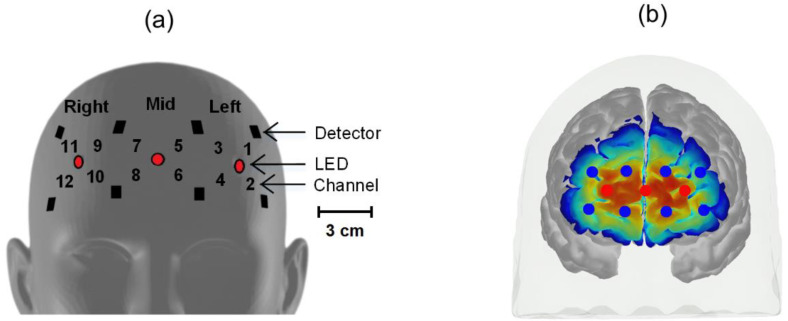
(**a**) Photodetector placement and channel positions. All the distances between the detector and LED are the same, 3 cm. The distances between the adjacent detectors are 5.5 cm horizontally and 4.5 cm vertically. Similarly, the distance between adjacent LEDs is 5.5 cm. (**b**) Sensitivity map for the depicted channel arrangement.

A wearable wireless fNIRS system developed at the Human Cyber-Physical Systems Lab at Florida International University was used for data acquisition in the experiment. The system architecture was based on the sensor system developed in [36,37,38] and modified to accommodate more channels required to sense the whole forehead. The improved system consists of three light-emitting diodes (LED) as a source of near-infrared (NIR) light, capable of multiwavelength (770 and 850 nm) emission, and eight photodetectors (PD) as light detectors, where the source–detector distance is 3 cm, with 0.3 cm variability. The differential path length (DPF) was 6.2 cm for 770 nm and 5.1 cm for 850 nm [39]. The LED and PD together form twelve channels for sensing, which are marked by channel numbers in Figure 3a. The sensitivity map for the depicted channel arrangement is presented in Figure 3b, derived from Homer2 Atlas Viewer [40,41]. The system covers the whole forehead for sensing, which is the region of interest (ROI) in this study. This ROI is subdivided into five sub-locations named Left, Mid, Right, Left-Mid and Right-Mid. Channels 1 to 4 sense the Left location on the forehead, Channels 5 to 8 sense the Mid location on the forehead, and the remaining Channels 9 to 12 sense the Right location on the forehead. The locations stated as Left-Mid and Right-Mid consist of Channels 1 to 10 and Channels 3 to 12, respectively. The location name for the whole forehead sensing area is Whole in subsequent descriptions. Each channel was sampled at a 25 Hz sampling rate. Additionally, the headband that houses the system is equipped with a nine-channel inertial measurement unit (IMU) and records the movement data concurrently with NIR data. IMU data were checked immediately after each session from each subject for movements during the recording, and the sessions that showed movements were discarded. The raw NIR signals were low-pass filtered with a third-order Butterworth bandpass filter with a 0.01–0.5 Hz cut-off frequency [42], and afterward, a two-second windowed moving-average filter was applied to further remove any physiological interference in the detected NIR signal, such as Mayer waves, respiration and heart rate [43,44]. Afterward, the modified Beer–Lambert [45] law was applied to convert the multiband raw NIR signal to oxygenation change signals, known as the change in oxygenated hemoglobin (ΔHbO2) and deoxygenated hemoglobin (ΔHbR).

To study how the workload detection accuracy varied with the length of sensing period along with the sensing location on the forehead, the oxygenation change signals were segmented with various window lengths, such as 5, 10, 20, 25 and 48 s [7]. There was about 50% overlap [7] in the windowing when the segmentation window length was less than the whole period of the 2-back or Rest state. The overlapping is assumed to be necessary to reduce inter-subject variability in the statistical temporal features of the signal [7,46]. After segmentation, each signal segment was labelled as 2-back or Resting-state accordingly.

This segmentation process resulted in n segments of 2-back state signal and m segments of resting-state signals. Here the values of n and m were dependent on the segmentation window lengths used in this study. For each session of any subject, it resulted in (n, m) = (1, 11) when segmenting with a 5 s window, (n, m) = (9, 5) for a 10 s window, (n, m) = (4, 2) for a 20 s window, (n, m) = (2, 1) for a 25 s window and (n, m) = (1, 1) when using the whole period of each state. These values of n and m also signify the number of samples in each class at different segmentation window lengths. All sessions of each subject, after segmentation and appropriate class-label assignment, resulted in 290, 140, 60, 30 and 20 samples for the segmentation window lengths of 5, 10, 20 and 25 s and the whole state period, respectively.

### 2.4. Feature Extraction

From each segmented hemodynamic change signal sample, commonly used statistical features were extracted, such as the mean [47,48], variance [49], slope [47,48] of polynomial fit, skewness [49,50], kurtosis [49,50] and correlation [7] of ΔHbO2 and ΔHbR. The extraction of these features from each channel resulted in a total of 11 features per channel for any samples under any segmentation window length. As there were various numbers of channels involved in sensing different locations on the forehead, various numbers of features resulted from each sample depending on the sensing location on the forehead. For example, there were 44 features for the Left, Mid and Right locations, whereas there were 110 features for the Left-Mid and Right-Mid locations for any length of segmentation. As there were 12 channels involved in the whole forehead location, there were 132 features recorded in the location denoted by Whole. The supplementary file to this article contains all the feature values segregated according to different sensing locations and segmentation window lengths. 

### 2.5. Feature Selection and Classification

In the case of the fNIRS-based classification problem, several classification methods have been used, such as Discriminant analysis, Support Vector Machine (SVM), Artificial Neural Network (ANN) and so on [50,51,52,53,54,55,56]. As the pivotal point of the study was to assess the cognitive workload monitoring accuracy variability using wearable fNIRS at various sensing locations on the forehead, the speed of the classifier was a crucial property for consideration. Thus, the Linear SVM, which had already been used in several other fNIRS-based classification studies [7,52,57], was selected as a classifier in this study. In this respect, the generic implementation of Linear SVM in the MATLAB platform with a box constraint of 1 and auto kernel scale was used for classification. For feature selection, two algorithms were used, namely, the Sequential Forward Selection (SFS) Wrapper algorithm [58] for feature subset selection and Relief algorithm [59,60]. Each of these algorithms has its own merits with respect to the statistical relevance (Relief) of the features to the classes [59] and the interaction of the training feature set (SFS) with the classifier algorithm [58]. Thus, both of the selected feature sets returned by these two algorithms were individually used in the classification using the linear SVM. The classification with the best accuracy among these two classification results was used in the statistical analysis. In this respect, as the SFS claims to return an optimal feature subset by heuristic search, the whole subset of the returned features was used in classification. On the other hand, as the Relief algorithm instead returns the ranks and weights for all the features, only the features with positive weights were used in the classification.

In the classification process, ten-fold testing cross-validation was applied [61]. In other words, there were 240 datasets resulting from segmentation, and in each of the classification processes on these datasets, the dataset was partitioned into ten subsets using a random selection of the observations for each partition. Then, the SVM was trained using the nine subsets of this dataset, leaving out the remaining one subset for testing. This leave-out subset is never seen by the classifier during the training phase. The training of the SVM was performed using ten-fold training cross-validation using those nine subsets of the partitioned data. Afterward, the trained SVM classifier was used to test the classification accuracy using the subset not used in training. Subsequently, this same training and testing procedure was applied on the other remaining nine subsets of this dataset in a nested cross-validation. The final accuracy of the classification on this dataset was calculated as the mean of these ten testing accuracies.

### 2.6. Statistical Analysis

For all the eight subjects, cognitive workload classification accuracies were calculated for the sensing locations of Left, Mid, Right, Left-Mid, Right-Mid and Whole, under several segmentation windows. The mean classification accuracies for each location across all the subjects were different from each other. Thus, to find the statistical significance of the differences in the mean classification accuracies for all subjects for each location (Left, Mid, Right, Left-Mid and Right-Mid) from the accuracy for the whole forehead location (Whole), a two-sample *t*-test was applied with a 5% significance level, which decided whether the means were statistically equal or not at that significance level.

## 3. Results and Discussion

All the classification accuracies from all the datasets are presented in Table 1. For the 5 s segmentation window, the lowest mean accuracy for all the subjects was 83.4%, with a standard deviation of 6.7%, which occurred when only the Right location was used. On the other hand, the highest mean accuracy occurred when the Whole forehead dataset was used to classify, which was 94.0%, with a 3.9% standard error. Like in the 5 s segmentation window, the Right location in all the other segmentation windows also resulted in the lowest classification accuracies, which were 84.0%, 86.5%, 90.8% and 95.0% for the 10, 15, 20, 25 and 48 s window lengths, respectively. For the 48 s segmentation window, the Left location also resulted in the lowest classification accuracy, like the Right location, but with a higher standard deviation, which was 6.5%. In the case of the highest mean accuracy of classifications beyond the 5 s segmentation window length, the Left-Mid location yielded the highest mean accuracy for the 20, 25 and 48 s segmentation window lengths, which were 93.6%, 95.9 and 98.3% respectively, and for the remaining 10 s segmentation window length, Right-Mid resulted in the highest mean accuracy of classification, which was 93.0%. The other details of the classification process, such as the F1 score, sensitivity, specificity and precision are listed in the table in Appendix A for each classification performed in this study. Similar to the mean classification accuracy results from the calculation process mentioned before, the classifier details described in Appendix A were also calculated.

Regarding the statistical analysis aforementioned, the two-sample *t*-test was used to test the statistical significance of the differences in the mean classification accuracies for each location (Left, Mid, Right, Left-Mid and Right-Mid) from that for the whole forehead location, denoted by Whole. The t-test hypothesis-testing decisions indicated that the mean classification accuracies were statistically different from the mean classification accuracy of the location Whole in the case of only the 10 and 20 s segmentation window lengths for location Right and the 5 s segmentation window length for locations Left and Right. In other words, except for these four cases, the mean classification accuracy for any location or segmentation window lengths, statistical significance was not found for the difference in the mean classification accuracies. Moreover, these results indicate that the Mid location, which is one of the three smallest sensing locations (Left, Mid, Right), resulted in a mean classification accuracy with a statistically insignificant difference, compared to the largest location Whole for any segmentation window lengths.

**Figure 4:**
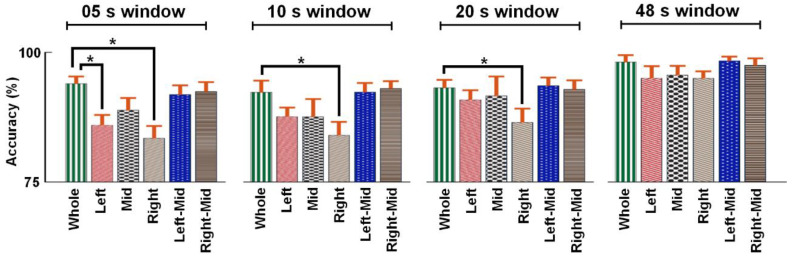
Mean accuracy of classifications across various location for different segmentation window lengths. The standard errors of the mean classification accuracies are presented by the error bars. The four classification accuracy means whose differences are statistically significant are highlighted for significance with stars.

The mean classification accuracies are depicted by the bar plot in Figure 4 for a qualitative assessment of how the classification accuracies varied along with the change in the sensing location on the forehead, which are grouped into segmentation windows. The error bars in the plot show the standard errors of the mean accuracies, which were estimated by dividing the standard deviation by the square root of the number of subjects in this study. From this plot, it is apparent that the differences in the mean accuracies between the largest location Whole and the other smaller locations were reduced with the increase in the segmentation window length.

Optical methods, such as fNIRS, which can reach a depth of a few centimeters only from the skin to the human brain, could only sense hemodynamic changes of the brain tissue that are closer to the cerebral surface, such as the PFC under the forehead. The PFC is one of the most functionally correlated subsystems [62] among other cerebral subsystems such as the Hippocampal Formation (HF), Inferior Parietal Lobule (IPL) and so on, which form the default network with respect to cognitive states. Moreover, research based on other methods such as fMRI and Positron Emission Tomography (PET) showed that the Medial PFC in this network is the most involved region of the PFC related to the cognitive states [63]. In this study, the n-back task was used to induce cognitive workload, which entails the involvement of the brain function named working memory. However, the outcome of any task execution by the human brain is accompanied by the other parts of human cognition such as memory retrieval, relational reasoning and multitasking behaviors. Other studies suggest that the frontopolar cortex (FPC), which we assumed to be sensed by the Mid location depicted in this study, is responsible for carrying out these parts of human cognition [64,65,66,67]. In congruence with those physiological study-based findings, the statistical results presented in this study also showed that sensing the Mid location only can result in significant accuracy in cognitive workload classification compared to using the location Whole on the forehead. Thus, from the system design point of view, the fNIRS system could be minimized to sense only the Mid location on the forehead with a statistically insignificant compromise of the cognitive workload detection accuracy. Similarly to the Mid location, the other two smallest sensing locations, Left and Right, could also be targeted for cognitive workload detection using a minimized fNIRS system but with higher latencies for obtaining significantly comparable accuracy.

## 4. Conclusions

The fNIRS is a promising modality for the ubiquitous monitoring of human cognitive workload. For the potential deployment of fNIRS, device design optimization regarding sensor position to boost wearability toward this aim of cognitive workload tracking is essential. In this respect, statistically significant classification accuracy for cognitive workloads can be achieved by sensing the Mid location on the human forehead rather than the entire forehead using fNIRS. This finding can be utilized by researchers to optimize their wearable wireless fNIRS systems, which are resource and power constrained by nature, as well as user comfort being a concern. While the purpose of this study was to investigate how the cognitive workload monitoring accuracy varied across the sensing locations on the forehead, the variability arising from the human subjects was assumed to be minimal, and the number of participants in this study was decided by considering common practice in other relevant studies. Future studies may investigate whether there are any subject-dependent variabilities in sensor location optimization for wearable fNIRS system design. The extracerebral tissue layer signal interferences were assumed to be minimal and similar in both the 2-back and resting-state signals. A future study may utilize a short separation channel to investigate these interferences. In this study, only the immobile state of the subject was considered. Thus, another promising future direction might be to investigate the effect of motion on sensor location optimization.

## Figures and Tables

**Table 1:** Classification accuracies along with means and standard deviations (SDs) across all subjects for each location on the forehead.

		Classification Accuracy (%)					
		Left	Mid	Right	Left-Mid	Right-Mid	Whole
5 s window	Sub1	82.4	88.3	84.5	89.3	89.0	92.1
	Sub2	87.2	95.5	80.0	98.3	97.6	98.6
	Sub3	80.0	87.2	72.1	87.2	90.0	91.4
	Sub4	84.1	76.9	85.2	83.8	85.2	89.3
	Sub5	94.1	92.1	93.1	96.9	99.3	98.6
	Sub6	91.4	97.9	87.6	96.2	96.9	98.3
	Sub7	90.0	88.9	87.9	92.4	93.4	93.1
	Sub8	78.3	84.1	77.2	90.7	88.3	90.3
	Mean ± SD	85.9 ± 5.7	88.9 ± 6.6	83.4 ± 6.7	91.9 ± 5.0	92.5 ± 5.1	94.0 ± 3.9
10 s window	Sub1	82.9	92.1	85.0	91.4	89.3	91.4
	Sub2	88.6	94.3	90.7	98.6	93.6	99.3
	Sub3	79.3	70.7	72.1	86.4	89.3	81.4
	Sub4	89.3	75.0	80.0	86.4	90.7	85.7
	Sub5	94.3	91.4	96.4	97.1	99.3	97.9
	Sub6	91.4	97.9	85.0	97.1	97.9	98.6
	Sub7	90.7	92.2	82.2	93.6	94.4	92.7
	Sub8	84.3	87.1	80.7	87.9	90.0	91.4
	Mean ± SD	87.6 ± 5.0	87.6 ± 9.6	84.0 ± 7.3	92.3 ± 5.0	93.0 ± 3.9	92.3 ± 6.3
20 s window	Sub1	86.7	95.0	83.3	88.3	90.0	93.3
	Sub2	93.3	100.0	93.3	98.3	95.0	95.0
	Sub3	83.3	81.7	70.0	86.7	85.0	85.0
	Sub4	93.3	70.0	86.7	91.7	93.3	90.0
	Sub5	98.3	98.3	93.3	98.3	100.0	100.0
	Sub6	96.7	100.0	90.0	96.7	96.7	93.3
	Sub7	86.6	94.7	85.2	95.2	94.7	95.2
	Sub8	88.3	93.3	90.0	93.3	88.3	93.3
	Mean ± SD	90.8 ± 5.4	91.6 ± 10.5	86.49 ± 7.6	93.6 ± 4.4	92.9 ± 4.8	93.2 ± 4.3
25 s window	Sub1	100.0	93.3	83.3	96.7	100.0	100.0
	Sub2	96.7	100.0	96.7	100.0	100.0	100.0
	Sub3	90.0	83.3	76.7	83.3	80.0	83.3
	Sub4	93.3	86.7	90.0	93.3	93.3	90.0
	Sub5	100.0	100.0	100.0	100.0	100.0	96.7
	Sub6	86.7	100.0	93.3	100.0	100.0	100.0
	Sub7	96.7	96.7	96.7	97.5	96.7	93.3
	Sub8	93.3	90.0	90.0	96.7	93.3	90.0
	Mean ± SD	94.6 ± 4.7	93.8 ± 6.5	90.8 ± 7.7	95.9 ± 5.6	95.4 ± 6.9	94.2 ± 6.1
48 s window	Sub1	100.0	100.0	90.0	100.0	100.0	100.0
	Sub2	95.0	100.0	95.0	100.0	100.0	100.0
	Sub3	85.0	90.0	90.0	95.0	90.0	90.0
	Sub4	100.0	90.0	95.0	100.0	95.0	100.0
	Sub5	100.0	100.0	100.0	100.0	100.0	100.0
	Sub6	85.0	100.0	95.0	100.0	100.0	100.0
	Sub7	95.0	90.0	100.0	96.7	95.0	95.0
	Sub8	100.0	95.0	95.0	95.0	100.0	100.0
	Mean ± SD	95 ± 6.5	95.6 ± 5.0	95 ± 3.8	98.3 ± 2.4	97.5 ± 3.8	98.1 ± 3.7

## Supplementary Materials

The following are available online at https://www.mdpi.com/1424-8220/20/18/5082/s1. Data file D1: feature values.

## Appendix A

Details of Classifiers

**Table A1:** Details of Classifiers (**Subjects** 1–4).

		Subject 1					Subject 2					Subject 3					Subject 4				
		Accuracy	F1 Score	Sensitivity	Specificity	Precision	Accuracy	F1 Score	Sensitivity	Specificity	Precision	Accuracy	F1 Score	Sensitivity	Specificity	Precision	Accuracy	F1 Score	Sensitivity	Specificity	Precision
05 second window	Left	82.4	86	89	72	84	87.2	90	94	75	86	80	89.8	87	87	93	84.1	88	89	75	86
	Mid	88.3	90	91	80	89	95.5	99	99	96	98	87	89.8	87	87	93	76.9	88	89	75	86
	Right	84.5	90	91	80	89	80.0	99	99	96	98	72	80.1	89	44	73	85.2	88	89	75	86
	Left-Mid	89.3	92	94	81	89	98.3	99	99	96	98	87	89.8	87	87	93	83.8	88	89	75	86
	Right-Mid	89.0	91	93	82	90	97.6	99	99	96	98	90	89.8	87	87	93	85.2	88	89	75	86
	ALL	92.1	90	91	80	89	98.6	99	99	96	98	91	89.8	87	87	93	89.3	92	94	82	90
10 second window	Left	82.9	94	97	82	91	88.6	99	100	96	98	79	89.0	83	92	95	89.3	90	92	76	88
	Mid	92.1	94	97	82	91	94.3	99	100	96	98	71	79.9	89	38	73	75.0	90	92	76	88
	Right	85.0	94	97	82	91	90.7	99	100	96	98	72	89.0	83	92	95	80.0	86	92	58	80
	Left-Mid	91.4	94	97	82	91	98.6	99	100	96	98	86	89.0	83	92	95	86.4	90	92	76	88
	Right-Mid	89.3	94	97	82	91	93.6	99	100	96	98	89	89.0	83	92	95	90.7	90	92	76	88
	ALL	91.4	94	97	82	91	99.3	99	100	96	98	81	89.0	83	92	95	85.7	90	92	76	88
20 second window	Left	86.7	92	93	75	91	93.3	99	100	95	98	83	92.1	100	60	85	93.3	94	95	85	94
	Mid	95.0	92	93	75	91	100.0	99	100	95	98	82	92.1	100	60	85	70.0	94	95	85	94
	Right	83.3	92	93	75	91	93.3	99	100	95	98	70	80.4	85	40	76	86.7	94	95	85	94
	Left-Mid	88.3	93	95	75	91	98.3	99	100	95	98	87	92.1	100	60	85	91.7	94	95	85	94
	Right-Mid	90.0	92	93	75	91	95.0	99	100	95	98	85	92.1	100	60	85	93.3	94	95	85	94
	ALL	93.3	92	93	75	91	95.0	99	100	95	98	85	92.1	100	60	85	90.0	94	95	85	94
25 second window	Left	100.0	97	95	100	100	96.7	100	100	100	100	90	90.6	95	60	87	93.3	97	100	80	93
	Mid	93.3	97	95	100	100	100.0	100	100	100	100	83	90.6	95	60	87	86.7	97	100	80	93
	Right	83.3	97	95	100	100	96.7	100	100	100	100	77	90.6	95	60	87	90.0	97	100	80	93
	Left-Mid	96.7	97	95	100	100	100.0	100	100	100	100	83	90.6	95	60	87	93.3	97	100	80	93
	Right-Mid	100.0	97	95	100	100	100.0	100	100	100	100	80	90.6	95	60	87	93.3	97	100	80	93
	ALL	100.0	97	95	100	100	100.0	100	100	100	100	83	90.6	95	60	87	90.0	97	100	80	93
48 second window	Left	100.0	100	100	100	100	95.0	100	100	100	100	85	97.4	100	90	95	100.0	100	100	100	100
	Mid	100.0	100	100	100	100	100.0	100	100	100	100	90	97.4	100	90	95	90.0	100	100	100	100
	Right	90.0	100	100	100	100	95.0	100	100	100	100	90	97.4	100	90	95	95.0	100	100	100	100
	Left-Mid	100.0	100	100	100	100	100.0	100	100	100	100	95	97.4	100	90	95	100.0	100	100	100	100
	Right-Mid	100.0	100	100	100	100	100.0	100	100	100	100	90	97.4	100	90	95	95.0	100	100	100	100
	ALL	100.0	100	100	100	100	100.0	100	100	100	100	90	97.4	100	90	95	100.0	100	100	100	100

**Table A2:** Details of Classifiers (**Subjects** 5–8).

		Subject 5					Subject 6					Subject 7					Subject 8				
		Accuracy	F1 Score	Sensitivity	Specificity	Precision	Accuracy	F1 Score	Sensitivity	Specificity	Precision	Accuracy	F1 Score	Sensitivity	Specificity	Precision	Accuracy	F1 Score	Sensitivity	Specificity	Precision
05 second window	Left	94.1	98	97	96	98	91.4	97	97	95	97	90.0	94	94	90	95	78.3	83	83	70	82
	Mid	92.1	98	97	96	98	97.9	97	97	95	97	88.9	94	94	90	95	84.1	88	92	71	84
	Right	93.1	98	97	96	98	87.6	97	97	95	97	87.9	94	94	90	95	77.2	82	85	65	80
	Left-Mid	96.9	98	97	96	98	96.2	97	97	95	97	92.4	94	94	90	95	90.7	93	94	85	91
	Right-Mid	99.3	98	97	96	98	96.9	97	97	95	97	93.4	94	94	90	95	88.3	91	95	77	88
	ALL	98.6	98	97	96	98	98.3	97	97	95	97	93.1	94	94	90	95	90.3	93	96	81	89
10 second window	Left	94.3	96	96	92	96	91.4	94	94	86	93	90.7	96	97	87	94	84.3	87	91	66	84
	Mid	91.4	94	93	88	94	97.9	98	99	94	97	92.2	96	97	87	94	87.1	87	91	66	84
	Right	96.4	97	96	96	98	85.0	89	89	78	88	82.2	96	97	87	94	80.7	87	91	66	84
	Left-Mid	97.1	98	98	96	98	97.1	98	99	94	97	93.6	96	97	87	94	87.9	92	93	78	90
	Right-Mid	99.3	97	96	96	98	97.9	98	99	94	97	94.4	96	97	87	94	90.0	87	91	66	84
	ALL	97.9	97	96	96	98	98.6	98	99	94	97	92.7	96	97	87	94	91.4	87	91	66	84
20 second window	Left	98.3	99	98	100	100	96.7	98	98	95	98	86.6	97	95	95	98	88.3	96	98	85	94
	Mid	98.3	99	98	100	100	100.0	98	98	95	98	94.7	97	95	95	98	93.3	96	98	85	94
	Right	93.3	99	98	100	100	90.0	98	98	95	98	85.2	97	95	95	98	90.0	96	98	85	94
	Left-Mid	98.3	99	98	100	100	96.7	98	98	95	98	95.2	97	95	95	98	93.3	96	98	85	94
	Right-Mid	100.0	99	98	100	100	96.7	98	98	95	98	94.7	97	95	95	98	88.3	96	98	85	94
	ALL	100.0	99	98	100	100	93.3	98	98	95	98	95.2	97	96	95	98	93.3	96	98	85	94
25 second window	Left	100.0	100	100	100	100	86.7	92	95	70	90	96.7	98	97	90	100	93.3	98	100	90	97
	Mid	100.0	100	100	100	100	100.0	100	100	100	100	96.7	98	97	90	100	90.0	98	100	90	97
	Right	100.0	100	100	100	100	93.3	100	100	100	100	96.7	98	97	90	100	90.0	98	100	90	97
	Left-Mid	100.0	100	100	100	100	100.0	100	100	100	100	97.5	98	97	90	100	96.7	98	100	90	97
	Right-Mid	100.0	100	100	100	100	100.0	100	100	100	100	96.7	98	97	90	100	93.3	98	100	90	97
	ALL	96.7	100	100	100	100	100.0	100	100	100	100	93.3	98	97	90	100	90.0	95	100	70	90
48 second window	Left	100.0	100	100	100	100	85.0	100	100	100	100	95.0	97	95	90	100	100.0	90	90	100	90
	Mid	100.0	100	100	100	100	100.0	100	100	100	100	90.0	97	95	90	100	95.0	90	90	100	90
	Right	100.0	100	100	100	100	95.0	100	100	100	100	100.0	100	100	90	100	95.0	90	90	100	90
	Left-Mid	100.0	100	100	100	100	100.0	100	100	100	100	96.7	97	95	90	100	95.0	90	90	100	90
	Right-Mid	100.0	100	100	100	100	100.0	100	100	100	100	95.0	97	95	90	100	100.0	90	90	100	90
	ALL	100.0	100	100	100	100	100.0	100	100	100	100	95.0	97	95	90	100	100.0	90	90	100	90
